# Organ Procurement from Deceased Donors and its Impact on Organ Transplantation in Iran during the First Ten Years of Cadaveric Transplantation

**Published:** 2012-08-01

**Authors:** S. M. Kazemeyni, M. Aghighi

**Affiliations:** 1 *Department of Urology, Shariati Hospital, Tehran University of Medical Sciences, Tehran, Iran*

**Keywords:** Deceased donor, Organ procurement, Cadaveric transplantation

## Abstract

The Act of transplantation from deceased and dead-brain donors was ratified in the parliament in 2000. In the subsequent two years, few number of organs were procured from dead-brain patients and transplanted. Later on, a national network was established for organ procurement; units for recognizing brain death were established in Tehran and some other cities to provide the necessary infrastructure for organ transplantation from deceased and dead-brain donors.

In this report, we described the outcome of organ procurement from deceased and dead-brain donors after 10 years of its establishment in Iran. To do so, we collected data from some relevant published articles and also reports of the Ministry of Health released between 2001 and 2010.

By the year 2010, 3673 organs were harvested from deceased donors and transplanted. The rate of liver transplantation has increased rapidly from 16 cases in 2001 to 280 cases in 2010—almost 18 times. There were 554 cadaveric kidney transplantation in 2010; it comprised 19% of total kidney transplantations that is almost 8 times that in 2001. Over the study period, organ procurement has increased by 6-fold.

The rate of organ procurement from deceased and dead-brain donors has increased dramatically over the studied period. Considering the existing potentials for this scheme of organ procurement, it seems that improving the Iranian Network for Transplant Organ Procurement will lead to better results.

## INTRODUCTION

Cadaveric organ transplantation in Iran dates back to almost 50 years ago [1]. Renal transplantation has flourished after Islamic Revolution of Iran, particularly at the beginning of Iran-Iraq war; during that era transplants were mainly procured from living donors. Then, Iran was at war and for limited financial resources and hemodialysis equipment, establishing a transplantation program seemed more appropriate. On the other hand, procurement of organs from deceased people was illegal at that time, and kidney transplantation was merely limited to harvested kidneys that were imported from other countries (*e.g.*, South Africa, the Netherlands) [2]. Therefore, a controlled living unrelated donor (LURD) was adopted [3, 7]. The rate of renal transplantation has increased dramatically. Almost half of patients with chronic renal failure requiring renal replacement therapy were transplanted [3, 4]. Despite all these success, based on some ethical and religious grounds, expansion of transplantation was questioned by some scholars. Transplant specialists began negotiation with religious authorities to issue the necessary edicts for overcoming religious obstacles associated with transplantation. Imam Khomeyni opened the way by issuing the necessary religious decree (*fatva*), with the legislation on brain death and organ transplantation finally passed by the parliament in 2000 [1, 6]. In 1999, the Brain Death Protocol was developed at the Ministry of Health and Medical Education. Several patients received organs from dead-brain donors within next two years. Later on, in 2001, the executive regulation was adopted in the governmental board. Subsequently, a national network for organ procurement was established and its branches began to work in a number of cities.

One decade has been passed since this legislation. Herein, we describe success and failure of this movement and evaluate the overall impact of this legislation on organ transplantation in Iran. To do so, we reviewed data collected from publications and reports of the Ministry of Health and some articles on the procurement of organs published between 2001 and 2010 [8-14].

Design of the National Network for Organ Procurement

To have an optimal network for organ procurement, many important factors should be considered. The factors are shortage of available organs for donation; the value of a cadaver; equitable distribution of the procured organs; preventing organ trafficking, transplant tourism, and commercialism; establishing the necessary infrastructure and logistics for correct organ transfer; education; and advocating and developing the culture of organ donation after death.

In 2001, the management center for organ transplantation affiliated to the Ministry of Health and Medical Education that proposed establishment of the network for organ procurement, after reviewing the most effective programs in the world [5], proposed two main issues that were finally approved by the governmental board. The issues included articles that mandates all hospitals to inform certain procurement offices of the occurrence of brain death; and establishing the regulations on how the procured organs allocation to patients in need of transplantation.

Finally, in 2001, establishment of an organization, the so-called “Iranian Network for Transplant Organ procurement” (IRANTOP) was officially announced. Job description of the network includes preparing an managing a waiting list; selection of appropriate recipients; establishing appropriate setup for transfer of organs; supervising organ procurement units; supervising financial issues; advocating and developing the culture of organ donation after death; establishing a complete database of organ donors and recipients; and establishing a protocol for organ procurement and transplantation from deceased and dead-brain donors. The network has several subunits including brain death identification subunit; brain death confirmation team; organs harvesting team; ICU and team for care of cadaver; allocation committee; reference laboratory for tissue typing; and statistics and research team.

One of the most important parts of this network is organ procurement unit (OPU), but then, all provincial universities could not establish this unit. Therefore, they just established a brain death identification unit and in case they found a dead brain patient, they would contact a well-equipped center.

The proposal of IRANTOP and its job description were ratified unanimously by a resolution made in the 49th meeting of deans of medical universities in 2002 [8]. In the first two years of its establishment, seven OPUs were established in university hospitals. Most of provincial universities established brain death identification units. Also, a bulletin and a Web site for IRANTOP were launched. This movement made it possible to start cadaveric transplantation formally and at high pace; the rate of increase was so high that the center won the annual health award by the president in 2003.

In the years later, the management chart of the center in Ministry of Health was revised. This change decreased the administrative position of the center that ultimately hampered the functionality of the network. Nevertheless, OPUs and some other units of the network have continued their work.

The number of OPUs increased to 12 in 2004 but not all of them could continue their work—three of them were closed, yet two have been added to the network recently. The OPUs active during the studied period (2001-2010) include Tehran (Imam Khomeyni Hospital, Shariati Hospital, Daneshvari Hospital), Shiraz, Mashhad, Urmia, and Isfahan. The activity of OPUs in Tehran, Shiraz and Mashhad was higher than other centers.

Other components of IRANTOP have also had good activities over the study period. The number of harvesting teams increased, and thus the harvesting time decreased; the number and ability of coordinating teams increased; centralized organ allocation was established in Tehran, yet in other provinces it was independent; the number of ICU wards with experts for care of dead brain donor increased. Logistic facilities for proper transfer of transplanted organs and harvesting teams have also improved. New transplantation wards for liver, pancreas, heart, lungs, and kidneys have been established.

Organ Transplantation from Deceased Donor in 2001–2010

During 10 years of the legislation, organ procurement from deceased donors increased to four transplants per million population. This rate though is not comparable with that in developed countries, is good enough for countries in this region.

**Figure 1:**
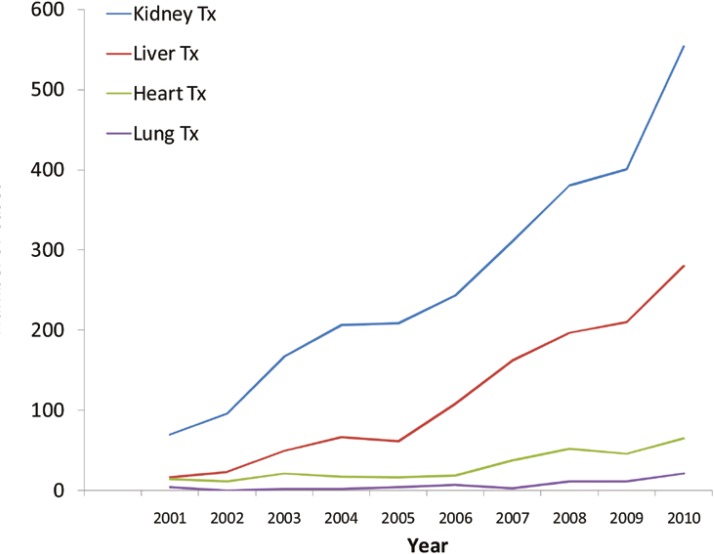
Number of cadaveric organ transplantations in Iran between 2001 and 2010

Most of the deceased donors were young [10-13]; the median age of donors was 29 (range: 6–63) years. Two-thirds of donors were male. Such an age and sex pattern might be attributed to the cause of brain death in Iran—*i.e.*, young men are more physically active and thus are more prone to accidents that result in more than half of the brain deaths. These characteristics—young and healthy—made their organs more favorable for transplantation. The rate of organ yield per donor was 3.06. This rate for organ procurement organizations in the United States was 3.13 (range: 2.63–3.89) [15]. The organs harvested include kidney, liver, heart, lung, pancreas, cornea and sometimes other tissues. Total number of organs harvested from deceased donors and transplanted in 2010 was 920 (Fig 1).

From 2001 to 2010, a total of 3673 organs was harvested from deceased donors and transplanted. Liver transplantation was mostly influenced by of the legislation of organ procurement from deceased donors. The rate of liver transplantation has had an 18-fold increase from 16 cases in 2001 to 280 cases in 2010 (Fig 1). The most active liver transplantation center is in Shiraz.

Cadaveric kidney transplantation rate has also had sharp increase (8-fold) from 70 cases in 2001 to 554 cases in 2010—19% of all kidney transplants. One of the most significant effects of organ procurement from deceased patients on kidney transplantation in Iran was a drop from 9% in 2001 to 4% in 2010 in the source of transplanted kidney from living related donors—the source has been shifted to deceased donors. Nevertheless, the ratio of living unrelated donors has not been decreased significantly. This is not necessarily a favorable effect and it would have been better if it had influenced the living unrelated donors. Other organs harvested from dead-brain donors during the study period included heart, lung and pancreas. However, their growth was not substantial as organ procurements were low. Pancreas transplantation has begun in Shiraz recently.

## CONCLUSION

The rate of organ procurement from deceased and dead-brain donors has increased dramatically over the first decade of passing the law on brain death. Considering the existing potentials for this scheme of organ procurement, it seems that improving the Iranian Network for Transplant Organ Procurement will lead to better results. The future of transplantation in Iran is promising. Transplantation, as an important therapeutic option, should be boosted in Iran by raising public awareness. Based on our successful experience, a regional organ procurement and transplantation network may be established.
